# Prevalence of multiple chronic conditions in New York State, 2011–2016

**DOI:** 10.1371/journal.pone.0211965

**Published:** 2019-02-07

**Authors:** Daniel Newman, Erica Levine, Sandeep P. Kishore

**Affiliations:** Arnhold Institute for Global Health at the Icahn School of Medicine at Mount Sinai, New York, NY, United States of America; Monash University, AUSTRALIA

## Abstract

**Introduction:**

To design effective policy and interventions, public health officials must have an accurate and granular picture of the state of multiple chronic conditions (MCC) in their region. The objective of this research is to describe the prevalence and distribution of MCC in New York State.

**Methods:**

We performed a secondary data analysis of the Behavioral Risk Factor Surveillance System (BRFSS) from 2011 through 2016 for New York adults (n = 76,186). We analyzed the self-reported prevalence of individuals having 0, 1, 2, or ≥ 3 chronic conditions by sex, race/ethnicity, age, health insurance type, annual household income, and whether respondents lived in New York City. We also examined the most common condition dyads and triads. Finally, we assessed the prevalence of MCC (2 or more chronic conditions) by county across New York State, and neighborhood within New York City.

**Results:**

During 2011–2016, 25.2% of adults in New York State had zero chronic conditions, 24.1% had 1 condition, 18.4% had 2 conditions, and 32.4% had 3 or more. The most prevalent dyad was hypertension and high cholesterol in 17.0% of individuals. The most prevalent triad was hypertension, high cholesterol, and arthritis in 4.5% of individuals. County prevalence of MCC ranged from 42.6% in Westchester County to 66.1% in Oneida County. The prevalence of MCC in New York City neighborhoods ranged from 33.5% in Gramercy Park—Murray Hill to 60.6% in High Bridge—Morrisania.

**Conclusion:**

This research contributes to the field’s understanding of multiple chronic conditions and allows policy and public health leaders in New York to better understand the prevalence and distribution of MCC.

## Introduction

As the United States population ages, the prevalence and burden of individuals living with multiple chronic conditions (MCC) are expected to rise.[1] By definition, chronic conditions are “conditions that last a year or more and require ongoing medical attention and/or limit activities of daily living,” and individuals with MCC are those living with two or more chronic conditions concurrently.[1] Because many chronic conditions share common modifiable (e.g. smoking, poor diet, physical inactivity) and non-modifiable (e.g. age) risk factors, efforts have increased to study MCC collectively rather than as individual, isolated conditions.[1–4]

Individuals with MCC face significant and unique challenges. MCC are associated with an increased risk of mortality and functional decline,[5,6] and adversely impact quality of life.[7] MCC also place a financial burden on individuals and the healthcare system, with estimates that each additional chronic condition can as much as double an individual’s overall healthcare expenditure.[8,9,10] Similarly, MCC have been shown to be associated with increased health resource utilization,[9] disproportionately high use of specialist services,[11] and more complex physician visits.[12]

In recognition of the rising importance of tackling MCC, the US Department of Health and Human Services (HHS) convened an interagency workgroup in 2011 to design a strategic framework for approaching MCC.[1] This framework encourages research that views chronic conditions collectively, rather than individually, and states objectives related to understanding the epidemiology of MCC and addressing disparities in MCC populations.[1]

Since the release of the HHS Strategic Framework, numerous research groups have examined the epidemiology of MCC. Researchers have estimated nationwide and state-by-state prevalence of MCC,[13–19] as well as the prevalence of MCC among specific population subgroups.[20–22] However, less research has been conducted on the distribution of MCC prevalence within states, and on the most common dyads (two-condition combinations) and triads (three-condition combinations) of chronic diseases.[18,23,24]

To design effective policy and interventions, public health officials must have an accurate and granular picture of the state of MCC in their region in addition to data on these conditions in isolation. This research attempts to create such a picture for New York by analyzing data from the Behavioral Risk Factor Surveillance Survey (BRFSS). Our main objectives were to: 1) estimate the prevalence of MCC for specific sociodemographic and geographic groups in New York State, and 2) define the most common dyads and triads (combinations of two and three conditions, respectively) for those sociodemographic and geographic groups.

## Methods

### Data

We used BRFSS, a publicly available data set collected each year by the Centers for Disease Control and Prevention (CDC) and state health agencies. BRFSS is a cross-sectional, state-based, telephone cell and landline telephone survey of the non-institutionalized adult population aged 18 years or older. Surveys are conducted in English and Spanish. Established in 1984 with 15 states, BRFSS now collects data in all 50 states as well as the District of Columbia and three U.S. territories.[25] BRFSS completes more than 400,000 adult interviews each year, making it the largest continuously conducted health survey system in the world.[25]

Our approach to using BRFSS in New York was adapted from a previously published analysis of BRFSS in Delaware.[24] We were interested in city and state trends of MCC that could provide context for our health system to support proactive interventions.

Data from 76,186 New York State adults surveyed from 2011 through 2016 were combined and analyzed. Combining multiple years of data was especially important for the county- and neighborhood-level analyses, in which prevalence estimates for several counties and neighborhoods were suppressed due to inadequate sample sizes following BRFSS data suppression guidelines.[24] Due to a significant methodology change in 2011, including a change to weighting methodology, BRFSS data before 2011 is not reliably comparable to data collected after 2011.[26,27] Questions were consistent across survey installments for every variable used in our analysis. BRFSS response rates for New York varied from 31.6% in 2013 to 36.3% in 2016. Sample sizes for New York ranged from 6,060 respondents in 2012 to 34,190 in 2016. This research was conducted using publicly available data and was exempt from Institutional Review Board review at the Icahn School of Medicine at Mount Sinai.

### Chronic condition variables

Our analysis included 12 self-reported conditions collected by BRFSS: arthritis, asthma, cancer, chronic obstructive pulmonary disease (COPD), depression, diabetes, heart disease, high blood pressure, high cholesterol, kidney disease, obesity, stroke. We included every chronic condition in the “Chronic Health Conditions” section of BRFSS. We also included obesity, which is collected as part of “Demographics”, and high blood pressure and high cholesterol, which are collected every odd-numbered year as part of a “Hypertension Awareness” module and a “Cholesterol Awareness” module, respectively.

BRFSS assesses the presence of chronic conditions using a variety of questions. Heart disease is assessed through separate questions on angina and myocardial infarction (heart attack), which are also reported as a calculated field that combines the two. For our analysis, we used the combined field as a single “heart disease” condition. Participants are asked separately whether they had ever had skin cancer or “any other types” of cancer. The two were combined into a single “cancer” in our analysis. The BRFSS question for COPD includes chronic obstructive pulmonary disease, emphysema and chronic bronchitis; arthritis includes arthritis, rheumatoid arthritis, gout, systemic lupus erythematosus, and fibromyalgia; depression includes any depressive disorder, including depression, major depression, minor depression, or dysthymia.

The following conditions were assessed by asking participants, “Have you ever been told that you have…” arthritis, cancer, chronic obstructive pulmonary disease, depression, heart disease, high cholesterol, kidney disease, and stroke. Respondents are asked two questions about asthma: one about whether they had it as a child, and another about having a current asthma diagnosis. We included only current asthma in our prevalence estimates, consistent with reports by the New York State Department of Health.[19] For diabetes and high blood pressure, we excluded women who reported having these conditions only while pregnant. Finally, obesity was calculated from participants’ self-reported current height and weight, with obesity defined as a body mass index (BMI) of 30 kg/m^2^ or higher.

### Sociodemographic variables

We analyzed prevalence rates by age, sex, race/ethnicity, health insurance, and annual household income, as well as geography.

We grouped respondents by annual household income into categories of less than $25,000, $25,000 to $49,999, and $50,000 or more in accordance with the New York State Department of Health’s chronic condition reports using BRFSS.[28] Consistent with previous reporting,[24] we grouped health insurance categories into private coverage (employer or union; self-insured), public coverage (Medicare; Medicaid or other state program), other coverage (Tricare (formerly the Civilian Health and Medical Program of the Uniformed Services), VA, or Military; Alaska Native, Indian Health Service, Tribal Health Services; some other source), and uninsured.[24] For our analysis of prevalence, dyads, and triads of chronic conditions, we designated individuals as being inside New York City or outside New York City based on their zip code. For our analysis of MCC prevalence in New York State by county, we used county of residence as reported in BRFSS. For or analysis of MCC prevalence in New York City, we grouped zip codes into United Hospital Fund (UHF) Neighborhoods, which consist of 42 adjoining zip code areas used by the New York State Department of Health to approximate New York City Community Planning Districts.[29]

### Statistical methods

We counted the presence of each of the 12 chronic conditions and coded individuals as having 0, 1, 2, or “3 or more” chronic conditions to analyze the crude and age-adjusted weighted prevalence of each of these buckets for different population subgroups. Age-adjusted estimates were adjusted by the direct method to the 2010 New York State Census population.[30] Additionally, we collapsed those four buckets into two (0–1 conditions and 2 or more conditions) and performed a chi-squared test for significant differences in the prevalence of MCC, defined as 2 or more chronic conditions, between sociodemographic variables. We also generated weighted prevalence rates for the most common dyad (two condition) and triad (three condition) combinations for each sociodemographic subgroup. Finally, we evaluated the prevalence of MCC for each county in New York State and for each neighborhood in New York City. For all analyses we used the state-wide weighting provided by BRFSS. As of 2016, the New York State data includes a county-level weight; however, we chose to use the state-level weight for all analyses in order to combine data from 2011–2016. Missing data were deleted in a pairwise fashion. This was infrequent for the variables used: <1% of respondents were missing data for each chronic condition for the years they were collected, except for obesity (7% missing).

We used R (version 3.5.0, R Project for Statistical Computing, Vienna, Austria) for our statistical analysis,[31] and in particular the ‘survey’ package to account for the complex sample design of BRFSS when generating prevalence estimates, confidence intervals, and statistical tests.[32] We calculated descriptive estimates for subgroups defined by age, sex, race/ethnicity, insurance coverage, annual household income, and geography. To ensure adequate sample sizes in subgroup analyses, we followed standard BRFSS data suppression guidelines. [24]

## Results

### Prevalence of MCC by selected characteristics

During 2011–2016, 25.2% of adults in New York State had none of the 12 chronic conditions we analyzed, 24.1% had a single condition, 18.4% had two conditions, and 32.4% had three or more (Table 1A). The overall prevalence of MCC (2 or more chronic conditions) for New York adults during this time period was 50.8%. The prevalence of MCC increased significantly with age, with 2 or more chronic conditions reported in 26.9% of 18 to 44-year-olds, 58.2% of 45 to 64-year-olds, and 78.9% of individuals 65 years or older (p<0.001). The prevalence of MCC did not vary significantly between women (51.0%) and men (50.5%) (p = 0.65). MCC prevalence did vary significantly by race/ethnicity; multiracial, non-Hispanic individuals report having the highest prevalence of MCC (61.3%) and Other race only, non-Hispanic reporting the lowest prevalence (35.8) (p<0.001). Both non-Hispanic White (53.7%) and non-Hispanic Black (51.6%) individuals had higher rates of MCC than Hispanic individuals (45.0%) (p<0.001). Uninsured individuals reported the lowest prevalence of MCC (45.0%), and individuals with public insurance the highest (68.5%) (p<0.001). MCC prevalence was lower with higher household income, with those earning less than $25,000 per year reporting 61.9% prevalence, and those earning $50,000 or more reporting 43.9% prevalence (p<0.001). Individuals living outside (53.1%) of New York City had a higher prevalence of MCC than individuals living inside (46.9%) New York City (p<0.001).

**Table 1:** Prevalence of chronic conditions^a^ among New York adults by selected characteristics, behavioral risk factor surveillance system, 2011–2016.

	0 conditions, % (95% CI)	1 condition, % (95% CI)	2 conditions, % (95% CI)	3+ conditions, % (95% CI)
**A. Unadjusted**				
**Overall**	25.2 (24.4, 26.0)	24.1 (23.3, 24.8)	18.4 (17.7, 19.1)	32.4 (31.5, 33.2)
**Age**				
18 to 44	43.3 (41.7, 45.0)	29.8 (28.2, 31.3)	15.0 (13.8, 16.2)	11.9 (10.8, 13.0)
45 to 64	18.0 (17.0, 19.0)	23.8 (22.6, 24.9)	21.0 (19.9, 22.0)	37.2 (35.9, 38.5)
65 or older	6.5 (5.7, 7.2)	14.7 (13.6, 15.7)	19.8 (18.5, 21.0)	59.1 (57.6, 60.6)
**Sex**				
Male	24.9 (23.7, 26.1)	24.5 (23.3, 25.8)	18.8 (17.7, 19.9)	31.8 (30.5, 33.0)
Female	25.4 (24.3, 26.5)	23.6 (22.6, 24.6)	18.0 (17.2, 18.9)	32.9 (31.8, 34.0)
**Race/ethnicity**				
Hispanic	28.9 (26.6, 31.3)	26.0 (23.7, 28.3)	17.0 (15.1, 18.9)	28.0 (25.8, 30.2)
White—Non-Hispanic	22.8 (21.8, 23.7)	23.5 (22.6, 24.4)	19.4 (18.5, 20.2)	34.3 (33.4, 35.3)
Black—Non-Hispanic	23.2 (21.0, 25.5)	25.2 (22.7, 27.6)	17.2 (15.3, 19.2)	34.4 (32.0, 36.8)
Multiracial, Non-Hispanic	17.8 (11.7, 23.9)	21.0 (14.5, 27.5)	20.8 (13.5, 28.0)	40.5 (31.8, 49.2)
Other race only, Non-Hispanic	40.7 (36.7, 44.6)	23.5 (20.1, 26.8)	15.2 (12.4, 17.9)	20.7 (17.2, 24.1)
**Insurance**				
Private	25.0 (23.7, 26.4)	26.3 (25.0, 27.6)	20.3 (19.1, 21.4)	28.4 (27.1, 29.6)
Public	13.3 (11.5, 15.1)	18.1 (16.2, 20.1)	18.1 (16.4, 19.8)	50.4 (48.1, 52.8)
Other	16.5 (12.3, 20.7)	18.9 (14.9, 23.0)	15.5 (11.9, 19.0)	49.1 (43.7, 54.4)
None	31.6 (28.7, 34.5)	23.4 (20.8, 25.9)	17.7 (15.4, 20.1)	27.3 (24.7, 29.9)
**Annual Household Income**				
Less than $25,000	18.1 (16.5, 19.7)	20.0 (18.4, 21.6)	16.7 (15.3, 18.1)	45.2 (43.3, 47.1)
$25,000 to $49,999	22.8 (21.1, 24.6)	23.0 (21.2, 24.7)	18.9 (17.3, 20.5)	35.3 (33.4, 37.1)
$50,000 or more	29.5 (28.2, 30.7)	26.6 (25.5, 27.8)	19.2 (18.2, 20.2)	24.7 (23.6, 25.7)
**Geography**				
NYC	28.6 (27.2, 29.9)	24.5 (23.3, 25.8)	17.9 (16.8, 18.9)	29.0 (27.7, 30.4)
Outside NYC	23.1 (22.1, 24.1)	23.8 (22.8, 24.8)	18.7 (17.9, 19.6)	34.4 (33.4, 35.4)
**B. Age-adjusted**				
**Overall**	31.6 (30.8, 32.4)	26.2 (25.4, 27.0)	17.3 (16.6, 18.0)	25.0 (24.1, 25.9)
**Sex**				
Male	32.6 (31.5, 33.7)	25.8 (24.8, 26.8)	16.6 (15.8, 17.4)	25.0 (23.9, 26.1)
Female	30.6 (29.4, 31.8)	26.5 (25.3, 27.7)	17.8 (16.7, 18.9)	25.1 (23.8, 26.4)
**Race/ethnicity**				
Hispanic	31.1 (28.8, 33.4)	26.8 (24.5, 29.1)	16.5 (14.6, 18.4)	25.7 (23.5, 27.9)
White—Non-Hispanic	30.5 (29.5, 31.5)	26.2 (25.3, 27.1)	18.2 (17.3, 19.1)	25.2 (24.3, 26.1)
Black—Non-Hispanic	28.1 (25.9, 30.3)	27.7 (25.2, 30.2)	16.3 (14.4, 18.2)	27.9 (25.5, 30.3)
Multiracial, Non-Hispanic	20.2 (14.1, 26.3)	25.5 (19.0, 32)	19.4 (12.1, 26.7)	34.8 (26.1, 43.5)
Other race only, Non-Hispanic	43.4 (39.4, 47.4)	23.1 (19.7, 26.5)	14.6 (11.8, 17.4)	18.8 (15.3, 22.3)
**Insurance**				
Private	32.6 (31.3, 33.9)	27.4 (26.1, 28.7)	18.3 (17.1, 19.5)	21.6 (20.3, 22.9)
Public	20.7 (18.9, 22.5)	23.7 (21.8, 25.6)	17.6 (15.9, 19.3)	38.0 (35.7, 40.3)
Other	32.0 (27.8, 36.2)	26.9 (22.9, 30.9)	13.7 (10.1, 17.3)	27.4 (22.0, 32.8)
None	33.6 (30.7, 36.5)	23.7 (21.1, 26.3)	17.3 (15.0, 19.6)	25.5 (22.9, 28.1)
**Annual Household Income**				
Less than $25,000	24.2 (22.6, 25.8)	23.3 (21.7, 24.9)	16.3 (14.9, 17.7)	36.2 (34.3, 38.1)
$25,000 to $49,999	31.5 (29.8, 33.2)	26.0 (24.2, 27.8)	18.1 (16.5, 19.7)	24.4 (22.5, 26.3)
$50,000 or more	34.6 (33.3, 35.9)	27.5 (26.4, 28.6)	17.6 (16.6, 18.6)	20.3 (19.2, 21.4)
**Geography**				
NYC	34.2 (32.8, 35.6)	26.0 (24.8, 27.2)	16.7 (15.6, 17.8)	23.1 (21.8, 24.4)
Outside NYC	29.7 (28.7, 30.7)	26.2 (25.2, 27.2)	17.6 (16.8, 18.4)	26.4 (25.4, 27.4)

Abbreviation: CI, Confidence Interval

^a^ Arthritis, asthma, cancer, chronic obstructive pulmonary disease, depression, diabetes, heart disease, high blood pressure, high cholesterol, kidney disease, obesity, stroke

### Prevalence of condition dyads and triads

For adults in New York State, the most prevalent chronic condition dyad was high cholesterol and hypertension (17.0%) (Table 2). This was the most prevalent dyad for both men and women, as well as for all age groups, insurance types, income levels, and for individuals inside and outside of New York City. The second most common dyad overall was found to be obesity and arthritis (8.3%), which was also the second most prevalent for men and women, all income levels, and inside and outside New York City.

**Table 2:** Most prevalent chronic condition^a^ dyads among New York adults by selected characteristics, behavioral risk factor surveillance system, 2011–2016.

	Dyad	% (95% CI)
**Overall**	High Cholesterol / Hypertension	17.0 (16.5, 17.6)
	Obesity / Arthritis	8.3 (8.0, 8.6)
	Hypertension / Arthritis	7.4 (7.1, 7.7)
	High Cholesterol / Arthritis	6.8 (6.5, 7.1)
	Obesity / Hypertension	6.7 (6.4, 6.9)
	Depression / Arthritis	6.2 (5.9, 6.5)
	Obesity / High Cholesterol	5.7 (5.5, 6.0)
	Obesity / Depression	4.9 (4.7, 5.2)
	Diabetes / Arthritis	4.9 (4.7, 5.2)
	Obesity / Diabetes	4.6 (4.4, 4.9)
**Age**		
18 to 44	High Cholesterol / Hypertension	4.1 (3.5, 4.6)
	Obesity / Depression	3.9 (3.5, 4.2)
	Obesity / Hypertension	3.0 (2.6, 3.3)
	Obesity / Asthma	2.8 (2.5, 3.1)
	Depression / Arthritis	2.7 (2.4, 3.0)
45 to 64	High Cholesterol / Hypertension	22.8 (21.7, 23.8)
	Obesity / Arthritis	12.4 (11.8, 13.0)
	Obesity / Hypertension	9.7 (9.1, 10.3)
	High Cholesterol / Arthritis	9.6 (9.1, 10.2)
	Hypertension / Arthritis	9.5 (9.0, 10.1)
65 or older	High Cholesterol / Hypertension	38.9 (37.5, 40.3)
	Hypertension / Arthritis	23.4 (22.4, 24.4)
	High Cholesterol / Arthritis	20.0 (19.0, 20.9)
	Obesity / Arthritis	15.8 (14.9, 16.7)
	Cancer / Arthritis	14.8 (14.1, 15.5)
**Sex**		
Male	High Cholesterol / Hypertension	17.8 (16.9, 18.7)
	Obesity / Arthritis	7.2 (6.7, 7.6)
	Obesity / Hypertension	6.7 (6.2, 7.1)
	Hypertension / Arthritis	6.1 (5.7, 6.4)
	Obesity / High Cholesterol	5.8 (5.4, 6.2)
Female	High Cholesterol / Hypertension	16.3 (15.6, 17.0)
	Obesity / Arthritis	9.4 (9.0, 9.9)
	Hypertension / Arthritis	8.7 (8.3, 9.1)
	Depression / Arthritis	8.0 (7.6, 8.4)
	High Cholesterol / Arthritis	7.9 (7.6, 8.3)
**Race/ethnicity**		
Hispanic	High Cholesterol / Hypertension	15.1 (13.6, 16.5)
	Obesity / Arthritis	7.5 (6.7, 8.3)
	Depression / Arthritis	6.8 (6.0, 7.5)
	Obesity / Hypertension	6.1 (5.3, 6.8)
	Obesity / Depression	6.0 (5.3, 6.7)
White—Non-Hispanic	High Cholesterol / Hypertension	17.8 (17.1, 18.4)
	Obesity / Arthritis	9.0 (8.6, 9.3)
	Hypertension / Arthritis	8.4 (8.0, 8.7)
	High Cholesterol / Arthritis	8.2 (7.8, 8.5)
	Obesity / Hypertension	6.8 (6.4, 7.1)
Black—Non-Hispanic	High Cholesterol / Hypertension	18.3 (16.6, 20.0)
	Obesity / Hypertension	9.4 (8.4, 10.3)
	Obesity / Arthritis	9.4 (8.4, 10.3)
	Hypertension / Arthritis	7.9 (7.1, 8.8)
	Obesity / High Cholesterol	6.6 (5.8, 7.5)
Multiracial, Non-Hispanic	Obesity / Arthritis	15.2 (10.8, 19.7)
	High Cholesterol / Hypertension	15.0 (9.4, 20.6)
	Diabetes / Arthritis	10.5 (6.4, 14.6)
	Depression / Arthritis	10.4 (7.5, 13.3)
	Obesity / Diabetes	10.1 (5.9, 14.4)
Other race only, Non-Hispanic	High Cholesterol / Hypertension	13.3 (10.9, 15.7)
	Hypertension / Arthritis	4.5 (3.5, 5.5)
	Diabetes / Arthritis	4.2 (3.0, 5.4)
	High Cholesterol / Arthritis	4.0 (3.0, 5.1)
	High Cholesterol / Diabetes	3.9 (2.9, 5.0)
**Insurance**		
Private	High Cholesterol / Hypertension	16.7 (15.8, 17.7)
	Obesity / Arthritis	7.8 (7.3, 8.2)
	High Cholesterol / Arthritis	6.5 (6.0, 6.9)
	Hypertension / Arthritis	6.4 (5.9, 6.8)
	Obesity / Hypertension	6.2 (5.7, 6.7)
Public	High Cholesterol / Hypertension	26.5 (24.7, 28.3)
	Obesity / Arthritis	13.8 (12.9, 14.7)
	Hypertension / Arthritis	11.9 (11.0, 12.8)
	Depression / Arthritis	11.4 (10.6, 12.3)
	High Cholesterol / Arthritis	10.5 (9.6, 11.4)
Other	High Cholesterol / Hypertension	26.3 (21.9, 30.7)
	Obesity / Arthritis	11.6 (9.7, 13.6)
	Hypertension / Arthritis	11.4 (9.4, 13.4)
	High Cholesterol / Arthritis	10.3 (8.4, 12.2)
	Obesity / Hypertension	8.8 (6.6, 10.9)
None	High Cholesterol / Hypertension	11.6 (10.3, 13.0)
	Obesity / Hypertension	5.4 (4.6, 6.2)
	Obesity / Arthritis	4.9 (4.2, 5.6)
	Hypertension / Arthritis	4.5 (3.8, 5.1)
	Obesity / High Cholesterol	3.9 (3.3, 4.6)
**Income**		
Less than $25,000	High Cholesterol / Hypertension	20.7 (19.5, 21.9)
	Obesity / Arthritis	11.6 (10.9, 12.3)
	Depression / Arthritis	10.7 (10.0, 11.4)
	Hypertension / Arthritis	10.5 (9.8, 11.2)
	Obesity / Hypertension	9.0 (8.3, 9.7)
$25,000 to $49,999	High Cholesterol / Hypertension	18.8 (17.5, 20.2)
	Obesity / Arthritis	9.2 (8.5, 10.0)
	Hypertension / Arthritis	8.4 (7.7, 9.1)
	High Cholesterol / Arthritis	7.5 (6.9, 8.2)
	Obesity / Hypertension	7.3 (6.6, 8.0)
$50,000 or more	High Cholesterol / Hypertension	14 (13.2, 14.7)
	Obesity / Arthritis	6.6 (6.2, 7.0)
	Obesity / Hypertension	5.5 (5.1, 5.9)
	High Cholesterol / Arthritis	5.3 (4.9, 5.7)
	Obesity / High Cholesterol	5.1 (4.8, 5.5)
**Geography**		
NYC	High Cholesterol / Hypertension	16.5 (15.6, 17.4)
	Obesity / Arthritis	7.1 (6.6, 7.7)
	Hypertension / Arthritis	6.5 (6.1, 7.0)
	High Cholesterol / Arthritis	6.0 (5.6, 6.4)
	Obesity / Hypertension	5.8 (5.4, 6.3)
Outside NYC	High Cholesterol / Hypertension	17.3 (16.7, 18.0)
	Obesity / Arthritis	9.1 (8.7, 9.4)
	Hypertension / Arthritis	8.0 (7.6, 8.3)
	High Cholesterol / Arthritis	7.3 (7.0, 7.7)
	Obesity / Hypertension	7.2 (6.8, 7.5)

Abbreviation: CI, Confidence Interval

^a^ Arthritis, asthma, cancer, chronic obstructive pulmonary disease, depression, diabetes, heart disease, high blood pressure, high cholesterol, kidney disease, obesity, stroke

The most common triad of chronic conditions for New York State adults was high cholesterol, hypertension, and arthritis (4.5%) (Table 3). This was also the case for sex and for geography (inside vs. outside New York City) but varied within all other sociodemographic categories. For example, the most common condition triad for non-Hispanic black individuals was obesity, high cholesterol, and hypertension (4.7%), and for non-Hispanic multiracial individuals was obesity, diabetes, and arthritis (6.9%). The most common triad for 18 to 44-year-olds was obesity, depression, and arthritis (1.1%), and obesity, high cholesterol, and hypertension was the most common triad for uninsured individuals (2.5%) and individuals with household incomes of $50,000 or more (3.0%).

**Table 3:** Most prevalent chronic condition^a^ triads among New York adults by selected characteristics, behavioral risk factor surveillance system, 2011–2016.

	Triad	% (95% CI)
**Overall**	High Cholesterol / Hypertension / Arthritis	4.5 (4.3, 4.7)
	Obesity / High Cholesterol / Hypertension	3.7 (3.5, 3.9)
	Obesity / Hypertension / Arthritis	2.8 (2.6, 3.0)
	Obesity / Depression / Arthritis	2.6 (2.4, 2.8)
	High Cholesterol / Hypertension / Diabetes	2.5 (2.4, 2.7)
	Obesity / Diabetes / Arthritis	2.5 (2.4, 2.7)
	Obesity / High Cholesterol / Arthritis	2.3 (2.2, 2.5)
	High Cholesterol / Hypertension / Depression	2.0 (1.9, 2.2)
	Hypertension / Diabetes / Arthritis	1.9 (1.8, 2.1)
	Hypertension / Depression / Arthritis	1.9 (1.7, 2.0)
**Age**		
18 to 44	Obesity / Depression / Arthritis	1.1 (0.9, 1.3)
	Obesity / High Cholesterol / Hypertension	1.0 (0.8, 1.2)
	Obesity / Depression / Asthma	0.9 (0.8, 1.1)
	Depression / Asthma / Arthritis	0.9 (0.7, 1.0)
	Obesity / Hypertension / Depression	0.8 (0.7, 1.0)
45 to 64	High Cholesterol / Hypertension / Arthritis	5.7 (5.3, 6.2)
	Obesity / High Cholesterol / Hypertension	5.7 (5.2, 6.1)
	Obesity / Depression / Arthritis	4.1 (3.8, 4.5)
	Obesity / Hypertension / Arthritis	4.1 (3.8, 4.5)
	Obesity / High Cholesterol / Arthritis	3.6 (3.3, 3.9)
65 or older	High Cholesterol / Hypertension / Arthritis	15.0 (14.2, 15.9)
	High Cholesterol / Hypertension / Diabetes	6.8 (6.2, 7.3)
	Obesity / High Cholesterol / Hypertension	6.7 (6.1, 7.3)
	Obesity / Hypertension / Arthritis	6.4 (5.8, 6.9)
	High Cholesterol / Hypertension / Cancer	6.3 (5.7, 6.8)
**Sex**		
Male	High Cholesterol / Hypertension / Arthritis	3.8 (3.5, 4.1)
	Obesity / High Cholesterol / Hypertension	3.6 (3.3, 4.0)
	High Cholesterol / Hypertension / Diabetes	2.5 (2.2, 2.7)
	Obesity / Hypertension / Arthritis	2.2 (2.0, 2.5)
	Obesity / Diabetes / Arthritis	2.2 (2.0, 2.4)
Female	High Cholesterol / Hypertension / Arthritis	5.2 (4.9, 5.5)
	Obesity / High Cholesterol / Hypertension	3.7 (3.4, 3.9)
	Obesity / Hypertension / Arthritis	3.4 (3.1, 3.6)
	Obesity / Depression / Arthritis	3.4 (3.1, 3.7)
	Obesity / Diabetes / Arthritis	2.9 (2.6, 3.1)
**Race/ethnicity**		
**Hispanic**	High Cholesterol / Hypertension / Arthritis	3.5 (3.0, 4.0)
	Obesity / High Cholesterol / Hypertension	3.3 (2.8, 3.9)
	Obesity / Depression / Arthritis	3.1 (2.6, 3.7)
	High Cholesterol / Hypertension / Diabetes	3.0 (2.5, 3.5)
	High Cholesterol / Hypertension / Depression	2.6 (2.1, 3.0)
White—Non-Hispanic	High Cholesterol / Hypertension / Arthritis	5.1 (4.9, 5.4)
	Obesity / High Cholesterol / Hypertension	3.8 (3.5, 4.0)
	Obesity / Hypertension / Arthritis	3.0 (2.8, 3.2)
	Obesity / Depression / Arthritis	2.6 (2.4, 2.8)
	Obesity / High Cholesterol / Arthritis	2.6 (2.4, 2.8)
Black—Non-Hispanic	Obesity / High Cholesterol / Hypertension	4.7 (4.1, 5.4)
	High Cholesterol / Hypertension / Arthritis	4.3 (3.7, 4.9)
	Obesity / Hypertension / Arthritis	3.7 (3.1, 4.3)
	High Cholesterol / Hypertension / Diabetes	3.7 (3.1, 4.2)
	Obesity / Diabetes / Arthritis	3.2 (2.6, 3.8)
Multiracial, Non-Hispanic	Obesity / Diabetes / Arthritis	6.9 (3, 10.7)
	Obesity / Depression / Arthritis	5.8 (3.4, 8.2)
	Obesity / COPD / Arthritis	5.5 (1.9, 9.0)
	Obesity / Asthma / Arthritis	5.4 (1.9, 9.0)
	Obesity / Hypertension / Arthritis	4.9 (2.1, 7.7)
Other race only, Non-Hispanic	High Cholesterol / Hypertension / Arthritis	2.6 (1.8, 3.4)
	High Cholesterol / Hypertension / Diabetes	2.4 (1.6, 3.3)
	Obesity / High Cholesterol / Hypertension	1.8 (1.1, 2.5)
	Diabetes / Depression / Arthritis	1.8 (0.8, 2.8)
	Obesity / Diabetes / Arthritis	1.8 (1.0, 2.5)
**Insurance**		
Private	High Cholesterol / Hypertension / Arthritis	3.9 (3.6, 4.2)
	Obesity / High Cholesterol / Hypertension	3.5 (3.1, 3.8)
	Obesity / Hypertension / Arthritis	2.6 (2.3, 2.9)
	Obesity / High Cholesterol / Arthritis	2.3 (2.0, 2.6)
	Obesity / Diabetes / Arthritis	2.0 (1.8, 2.2)
Public	High Cholesterol / Hypertension / Arthritis	7.6 (6.8, 8.3)
	Obesity / Depression / Arthritis	5.2 (4.6, 5.8)
	Obesity / Diabetes / Arthritis	4.9 (4.4, 5.5)
	Obesity / High Cholesterol / Hypertension	4.9 (4.3, 5.5)
	Obesity / Hypertension / Arthritis	4.3 (3.7, 4.9)
Other	High Cholesterol / Hypertension / Arthritis	7.4 (5.8, 9.0)
	Obesity / High Cholesterol / Hypertension	5.5 (3.6, 7.4)
	High Cholesterol / Hypertension / Diabetes	5.3 (3.3, 7.3)
	Obesity / Hypertension / Arthritis	4.3 (3.0, 5.5)
	Obesity / Diabetes / Arthritis	4.2 (2.9, 5.5)
None	Obesity / High Cholesterol / Hypertension	2.5 (2.0, 3.1)
	High Cholesterol / Hypertension / Arthritis	2.2 (1.9, 2.6)
	Obesity / Hypertension / Arthritis	1.8 (1.4, 2.2)
	High Cholesterol / Hypertension / Diabetes	1.7 (1.4, 2.1)
	High Cholesterol / Hypertension / Depression	1.5 (1.1, 1.8)
**Income**		
Less than $25,000	High Cholesterol / Hypertension / Arthritis	6.3 (5.8, 6.8)
	Obesity / High Cholesterol / Hypertension	5.0 (4.5, 5.5)
	Obesity / Depression / Arthritis	4.9 (4.4, 5.4)
	Obesity / Hypertension / Arthritis	4.4 (3.9, 4.9)
	High Cholesterol / Hypertension / Diabetes	4.2 (3.8, 4.7)
$25,000 to $49,999	High Cholesterol / Hypertension / Arthritis	5.2 (4.7, 5.7)
	Obesity / High Cholesterol / Hypertension	4.0 (3.6, 4.5)
	Obesity / Hypertension / Arthritis	3.0 (2.6, 3.4)
	Obesity / Diabetes / Arthritis	2.8 (2.3, 3.2)
	High Cholesterol / Hypertension / Diabetes	2.7 (2.3, 3.1)
$50,000 or more	Obesity / High Cholesterol / Hypertension	3.0 (2.7, 3.3)
	High Cholesterol / Hypertension / Arthritis	2.9 (2.6, 3.1)
	Obesity / Hypertension / Arthritis	2.0 (1.8, 2.3)
	Obesity / High Cholesterol / Arthritis	1.8 (1.6, 2.0)
	Obesity / Depression / Arthritis	1.5 (1.3, 1.8)
**Geography**		
NYC	High Cholesterol / Hypertension / Arthritis	4.0 (3.7, 4.4)
	Obesity / High Cholesterol / Hypertension	3.2 (2.9, 3.5)
	High Cholesterol / Hypertension / Diabetes	2.8 (2.5, 3.2)
	Obesity / Hypertension / Arthritis	2.4 (2.1, 2.7)
	Obesity / Depression / Arthritis	2.3 (2.0, 2.7)
Outside NYC	High Cholesterol / Hypertension / Arthritis	4.8 (4.5, 5.0)
	Obesity / High Cholesterol / Hypertension	3.9 (3.7, 4.2)
	Obesity / Hypertension / Arthritis	3.1 (2.9, 3.3)
	Obesity / Depression / Arthritis	2.8 (2.6, 3.0)
	Obesity / Diabetes / Arthritis	2.7 (2.5, 2.9)

Abbreviation: CI, Confidence Interval

^a^ Arthritis, asthma, cancer, chronic obstructive pulmonary disease, depression, diabetes, heart disease, high blood pressure, high cholesterol, kidney disease, obesity, stroke

### Geographic distribution of MCC

**Fig 1:**
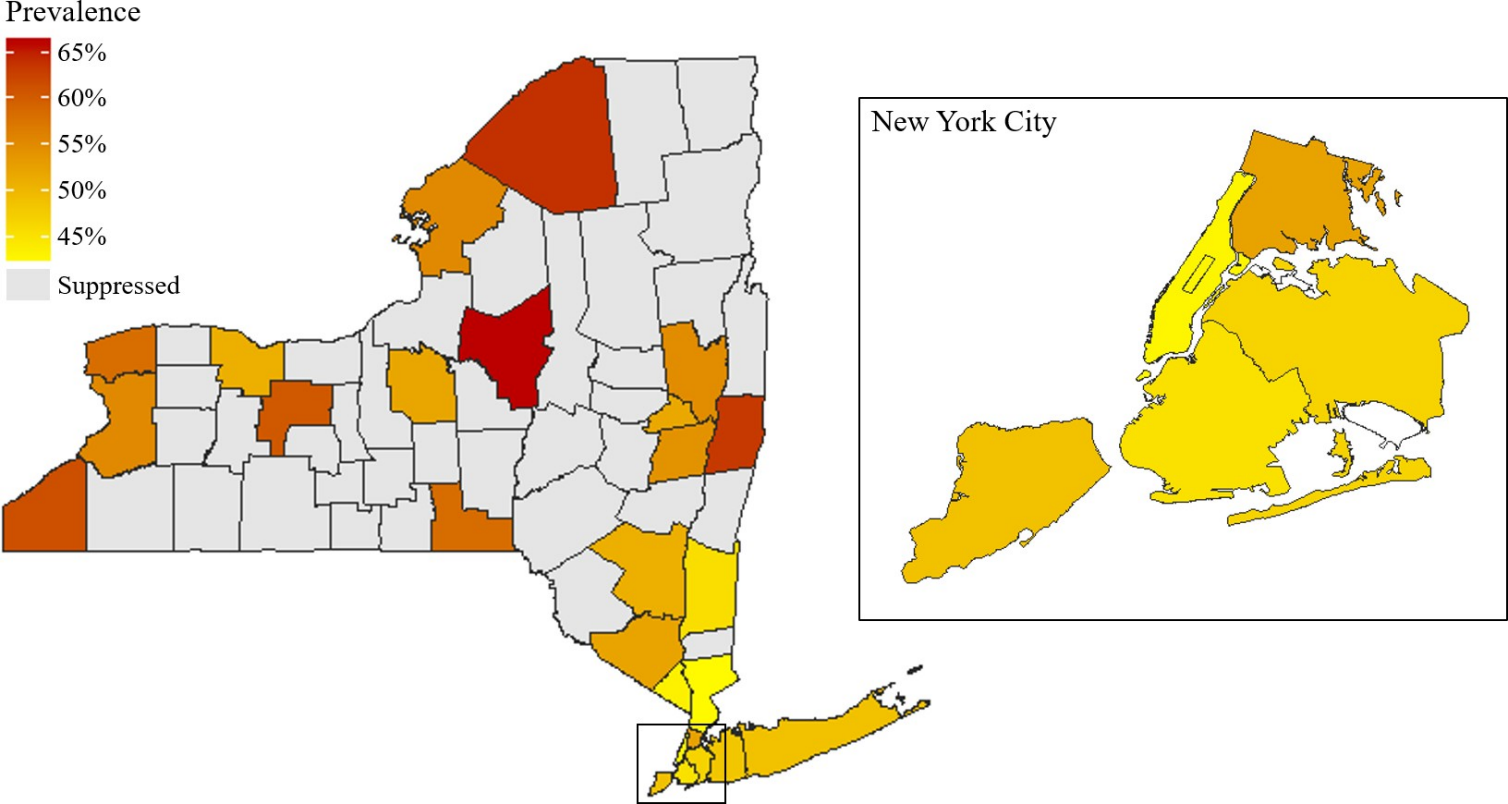
Prevalence of New York adults with two or more chronic conditions by county. Behavioral Risk Factor Surveillance System, 2011–2016.

The prevalence of MCC (2 or more chronic conditions) varied significantly between counties within New York State, and between neighborhoods within New York City. Fig 1 maps the prevalence of MCC by county for all of New York State, and S1 Table lists the data for this figure. County prevalence of MCC ranged from 42.6% in Westchester County to 66.1% in Oneida County. Of the 62 counties in New York State, prevalence estimates for 36 (58%) were suppressed based on BRFSS data suppression guidelines; these 36 counties account for roughly 11% of the state’s total population as of the 2010 census.[33] Of New York City boroughs, the Bronx had the highest prevalence of MCC (52.5%), followed by Staten Island (Richmond County) (48.9%), Queens (46.9%), Brooklyn (Kings County) (45.4%), and Manhattan (New York County) (43.1%).

**Fig 2:**
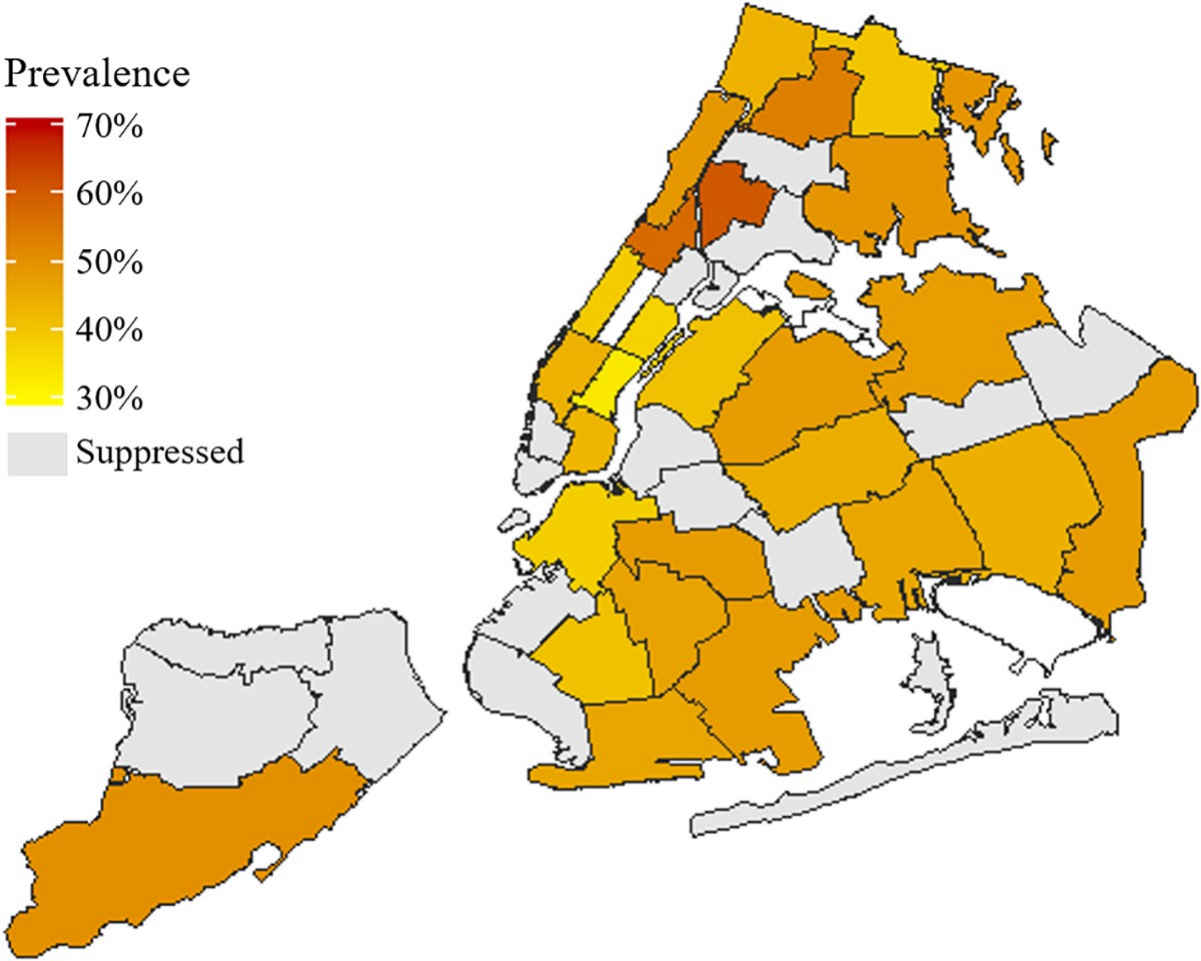
Prevalence of New York City adults with two or more chronic conditions by United Hospital Fund (UHF 42) neighborhood. Behavioral Risk Factor Surveillance System, 2011–2016.

The prevalence of MCC in New York City neighborhoods ranged from 33.5% in Gramercy Park—Murray Hill (Manhattan) to 60.6% in High Bridge—Morrisania (Bronx). Fig 2 maps the prevalence of MCC by county for all of New York State, and S2 Table lists the data for this figure. Of the 42 UHF neighborhoods in New York City, prevalence estimates for 16 (38%) were suppressed based on BRFSS data suppression guidelines; those neighborhoods account for roughly 36% of the city’s population as of the 2010 census.[34]

## Discussion

Nearly 51% of adults in New York State had MCC during 2011–2016 based on BRFSS data. These estimates are higher than previous estimates using NHIS data, which estimated the prevalence of MCC in New York State to be 21.3% in 2014.[13] This discrepancy in prevalence is likely due to differences in the number and type of chronic conditions included in each analysis. For example, the previous study using data from NHIS did not include high cholesterol, obesity, and depression, which had the highest (36.6%), 3rd highest (25.0%), and 5th highest (15.7%) individual prevalence rates respectively of the 12 conditions included in our analysis. High cholesterol and depression were both included in the 20 conditions identified by HHS for inclusion in studies of MCC, but are not reported in NHIS.[1,13,35]

MCC prevalence was generally higher in older adults, those with public insurance, and those with a lower household income. It was also generally lower among Hispanic individuals when compared to non-Hispanic white and black individuals. This distribution of MCC holds when adjusting for age (Table 1B), and is largely consistent with studies of MCC using national data and data from other states.[16,24,36] High blood pressure / high cholesterol and obesity / arthritis were the two most prevalent dyads in New York State, and arthritis / high blood pressure / high cholesterol was the most common triad. This is consistent with the results found in Delaware, with the order of the top two dyads reversed.[24]

The descriptive analysis presented here is limited in its ability to infer causality in regional variation of MCC prevalence; however, some possible drivers of regional variation have been elucidated in prior research and can be hypothesized here. Within New York City for example, we found the lowest prevalence of MCC in Gramercy Park—Murray Hill (Manhattan), and highest in High Bridge—Morrisania (Bronx). Gramercy Park—Murray Hill has an older population on average,[37, 38] which does not align with the positive correlation between age and MCC previously reported.[16–18, 24] This discrepancy may, however, be explained by vast differences in income between the two neighborhoods: 8% of the Gramercy Park—Murray Hill population lives below the poverty level, compared to 41% in High Bridge–Morrisania.[37, 38]

There were several limitations to this study. First, the BRFSS survey data are subject to the common biases of self-report surveys: underreporting due to recall bias and social desirability bias, as well as missing undiagnosed conditions. Other methodologies for estimating MCC prevalence, such as analyzing electronic health records, may mitigate limitations of surveys; however, these methods also introduce significant sampling bias. Second, BRFSS excludes people living in institutions, nursing homes, long-term care facilities, and correctional institutions, and so these results may differ from MCC estimates in these populations. Finally, our analysis is limited to the conditions chosen for inclusion in BRFSS. BRFSS excludes many of the 20 conditions identified by HHS for MCC research, including a number of mental health disorders (dementia, schizophrenia) and chronic viral diseases (HIV/AIDS, viral hepatitis).[1, 35] As a result our analysis likely underrepresents the true prevalence of MCC in New York State.

Despite these limitations, this research takes an important step forward in understanding the prevalence and distribution of MCC among New York adults. Future studies could build on this work by analyzing the impact of risk factors on MCC in New York State and by attempting to understand why differences between subgroups and geographies exist. Additionally, there are opportunities in the BRFSS data set to understand the impacts of MCC on quality of life and overall health. Future studies may also include comparisons between multiple states.

## Supporting information

S1 Table Prevalence of New York Adults with Two or More Chronic Conditions by County. Behavioral Risk Factor Surveillance System, 2011–2016.
(DOCX)

S2 Table Prevalence of New York City Adults with Two or More Chronic Conditions by United Hospital Fund (UHF 42) Neighborhood. Behavioral Risk Factor Surveillance System, 2011–2016.
(DOCX)

## References

[1] Multiple chronic conditions—a strategic framework: optimum health and quality of life for individuals with multiple chronic conditions. Washington, DC: US Department of Health and Human Services [Internet]. 2010;2 Available from: https://www.giaging.org/documents/mcc_framework.pdf

[2] US Department of Health and Human Services. HHS initiative on multiple chronic conditions. Available from: www.hhs.gov/ash/about-ash/multiple-chronic-conditions/index.html. Accessed December 14, 2018.

[3] Bauer UE, Briss PA, Goodman RA, Bowman BA. Prevention of chronic disease in the 21st century: elimination of the leading preventable causes of premature death and disability in the USA. Lancet. 2014;384(9937):45–52. 10.1016/S0140-6736(14)60648-6 24996589

[4] Voetsch K, Sequeira S, Chavez AH. A customizable model for chronic disease coordination: lessons learned from the Coordinated Chronic Disease Program. Prev Chronic Dis. 2016 3;13: E43 10.5888/pcd13.150509 27032986 PMC4825748

[5] Marengoni A, Von Strauss E, Rizzuto D, Winblad B, Fratiglioni L. The impact of chronic multimorbidity and disability on functional decline and survival in elderly persons. A community-based, longitudinal study. J Intern Med. 2009;265(2):288–95. 10.1111/j.1365-2796.2008.02017.x 19192038

[6] Newman AB, Boudreau RM, Naydeck BL, Fried LF, Harris TB. A physiologic index of comorbidity: relationship to mortality and disability. J Gerontol A Biol Sci Med Sci. 2008 6;63(6):603–9. 18559635 10.1093/gerona/63.6.603 PMC2496995

[7] Bayliss EA, Ellis JL, Steiner JF. Subjective assessments of comorbidity correlate with quality of life health outcomes: initial validation of a comorbidity assessment instrument. Health Qual Life Outcomes. 2005 9 1;3:51 10.1186/1477-7525-3-51 16137329 PMC1208932

[8] McPhail SM. Multimorbidity in chronic disease: impact on health care resources and costs. Risk Manag Healthc Policy. 2016 7 5;9:143–56. 10.2147/RMHP.S97248 27462182 PMC4939994

[9] Wolff JL, Starfield B, Anderson G. Prevalence, expenditures, and complications of multiple chronic conditions in the elderly. Arch Intern Med. 2002 11 11;162(20):2269–76. 12418941 10.1001/archinte.162.20.2269

[10] Machlin SR, Soni A. Health care expenditures for adults with multiple treated chronic conditions: estimates from the Medical Expenditure Panel Survey, 2009. Prev Chronic Dis. 2013 4 25;10:E63 10.5888/pcd10.120172 23618543 PMC3652712

[11] Anderson G. Chronic Care: Making the Case for Ongoing Care, Robert Wood Johnson Foundation 2010.

[12] Beasley JW, Hankey TH, Erickson R, Stange KC, Mundt M, Elliott M, et al How many problems do family physicians manage at each encounter? A WReN study. Ann Fam Med. 2004 9;2(5):405–10. 10.1370/afm.94 15506571 PMC1466713

[13] Ward BW, Black LI. State and Regional Prevalence of Diagnosed Multiple Chronic Conditions Among Adults Aged ≥18 Years—United States, 2014. MMWR Morb Mortal Wkly Rep. 2016 7 29;65(29):735–8. 10.15585/mmwr.mm6529a3 27467707

[14] Adams ML, Grandpre J, Katz DL, Shenson D. Linear association between number of modifiable risk factors and multiple chronic conditions: Results from the Behavioral Risk Factor Surveillance System. Prev Med. 2017 12;105:169–75. 10.1016/j.ypmed.2017.09.013 28917949

[15] Buttorff C, Ruder T, Bauman M. Multiple chronic conditions in the United States. Santa Monica (CA): RAND Corporation [Internet]. 2017; Available from: http://sbgg.org.br/informativos/29-06-17/1497877975_1_Chronic_Conditions.pdf

[16] Adams ML. Differences Between Younger and Older US Adults With Multiple Chronic Conditions. Prev Chronic Dis. 2017 9 7;14:E76 10.5888/pcd14.160613 28880839 PMC5590488

[17] Ward BW, Schiller JS, Goodman RA. Multiple chronic conditions among US adults: a 2012 update. Prev Chronic Dis. 2014 4 17;11:E62 10.5888/pcd11.130389 24742395 PMC3992293

[18] Ward BW, Schiller JS. Prevalence of multiple chronic conditions among US adults: estimates from the National Health Interview Survey, 2010. Prev Chronic Dis. 2013 4 25;10:E65 10.5888/pcd10.120203 23618545 PMC3652717

[19] Preventing Chronic Diseases and Supporting Health and Healthy Communities. New York State Department of Health, Division of Chronic Disease Prevention; 2017 Jan.

[20] Lochner KA, Goodman RA, Posner S, Parekh A. Multiple chronic conditions among Medicare beneficiaries: state-level variations in prevalence, utilization, and cost, 2011. Medicare Medicaid Res Rev [Internet]. 2013 7 23;3(3). Available from: 10.5600/mmrr.003.03.b02 PMC3983735 24753976

[21] Rezaee ME, Pollock M. Prevalence and Associated Cost and Utilization of Multiple Chronic Conditions in the Outpatient Setting among Adult Members of an Employer-Based Health Plan. Popul Health Manag. 2015 12;18(6):421–8. 10.1089/pop.2014.0124 25919016

[22] Clark NM, Lachance L, Benedict MB, Little R, Leo H, Awad DF, et al The extent and patterns of multiple chronic conditions in low-income children. Clin Pediatr. 2015 4;54(4):353–8. 10.1177/0009922815574073 25802420

[23] Machlin SR, Soni A. Health care expenditures for adults with multiple treated chronic conditions: estimates from the Medical Expenditure Panel Survey, 2009. Prev Chronic Dis. 2013 4 25;10:E63 10.5888/pcd10.120172 23618543 PMC3652712

[24] Gupta S. Burden of Multiple Chronic Conditions in Delaware, 2011–2014. Prev Chronic Dis. 2016 11 23;13:E160 10.5888/pcd13.160264 27880632 PMC5127174

[25] CDC—About BRFSS [Internet]. [cited 2018 Jul 30]. Available from: https://www.cdc.gov/brfss/about/index.htm

[26] Methodologic Changes in BRFSS in 2011 | SRC | CDC [Internet]. 2018 [cited 2018 Jul 30]. Available from: https://www.cdc.gov/surveillancepractice/reports/brfss/brfss.html

[27] United State Centers for Disease Control and Prevention. Comparability of Data: BRFSS 2011 National Center for Chronic Disease Prevention and Health Promotion, Division of Population Health. Behavioral Risk Factor Surveillance System 10.4236/health.2013.510A2002

[28] BRFSS Reports [Internet]. New York State Department of Health. [cited 2018 Aug 1]. Available from: https://www.health.ny.gov/statistics/brfss/reports/

[29] New York State Department of Health. NYC Neighborhood ZIP Code Definitions [Internet]. [cited 2018 Aug 2]. Available from: https://www.health.ny.gov/statistics/cancer/registry/appendix/neighborhoods.htm

[30] New York State Department of Health. Vital Statistics of New York State 2010 [Internet]. [cited 2018 Sep 4]. Available from: https://www.health.ny.gov/statistics/vital_statistics/2010/

[31] R Development Core Team. R: A language and environment for statistical computing. Vienna, Austria: R Foundation for Statistical Computing; 2008.

[32] Lumley T. Analysis of Complex Survey Samples. Vol. 9, Journal of Statistical Software. 2004 p. 1–19.

[33] U.S. Census Bureau. Annual Estimates of the Resident Population (New York State, by County): April 1, 2010 to July 1, 2017. 2010 Oct 5 [cited 2018 Aug 2]; Available from: https://factfinder.census.gov/faces/tableservices/jsf/pages/productview.xhtml?src=bkmk

[34] NYC Department of Health. NYC 2010 Census Query [Internet]. NYC Health. [cited 2018 Aug 2]. Available from: https://a816-healthpsi.nyc.gov/epiquery/Census/index2010.html

[35] U.S. Centers for Medicare & Medicaid Services. Chronic Conditions [Internet]. CMS.gov. 2017 [cited 2018 Aug 3]. Available from: https://www.cms.gov/Research-Statistics-Data-and-Systems/Statistics-Trends-and-Reports/Chronic-Conditions/CC_Main.html

[36] Freid VM, Bernstein AB, Bush MA. Multiple chronic conditions among adults aged 45 and over: trends over the past 10 years. NCHS Data Brief. 2012 7;(100):1–8. 23101759

[37] New York City Department of Health and Mental Hygiene. Community Health Profiles: Gramercy Park and Murray Hill [Internet]. 2006. Available from: https://www1.nyc.gov/assets/doh/downloads/pdf/data/2006chp-307.pdf

[38] New York City Department of Health and Mental Hygiene. Community Health Profiles: High Bridge and Morrisania [Internet]. 2006. Available from: https://www1.nyc.gov/assets/doh/downloads/pdf/data/2006chp-106.pdf

