# Supplementary material for: Prevalence of multiple chronic conditions in New York State, 2011–2016
Source: PLoS One. 2019 Feb 7;14(2):e0211965. doi: 10.1371/journal.pone.0211965 (PMC6366719; doi:10.1371/journal.pone.0211965)
Supplement: S1 Table — Behavioral Risk Factor Surveillance System, 2011–2016. (DOCX) [file pone.0211965.s001.docx]

# Supplementary Table 1. Prevalence of New York Adults with Two or More Chronic Conditions^a^ by County. Behavioral Risk Factor Surveillance System, 2011–2016.

| **County** | **% (95% CI)** |
| --- | --- |
| Albany County | 54.3 (47.7, 60.9) |
| Allegany County | Suppressed |
| Bronx County | 52.5 (48.8, 56.2) |
| Broome County | 58.5 (50.6, 66.5) |
| Cattaraugus County | Suppressed |
| Cayuga County | Suppressed |
| Chautauqua County | 61.4 (52.2, 70.7) |
| Chemung County | Suppressed |
| Chenango County | Suppressed |
| Clinton County | Suppressed |
| Columbia County | Suppressed |
| Cortland County | Suppressed |
| Delaware County | Suppressed |
| Dutchess County | 45.7 (38.1, 53.4) |
| Erie County | 55.4 (51.1, 59.6) |
| Essex County | Suppressed |
| Franklin County | Suppressed |
| Fulton County | Suppressed |
| Genesee County | Suppressed |
| Greene County | Suppressed |
| Hamilton County | Suppressed |
| Herkimer County | Suppressed |
| Jefferson County | 55.4 (46.1, 64.8) |
| Kings County | 45.4 (42.8, 48.0) |
| Lewis County | Suppressed |
| Livingston County | Suppressed |
| Madison County | Suppressed |
| Monroe County | 51.1 (46.7, 55.6) |
| Montgomery County | Suppressed |
| Nassau County | 48.4 (44.5, 52.3) |
| New York County | 43.1 (40.5, 45.6) |
| Niagara County | 58.3 (51.3, 65.3) |
| Oneida County | 66.1 (59.2, 73.0) |
| Onondaga County | 52.1 (46.4, 57.9) |
| Ontario County | 60.6 (51.0, 70.2) |
| Orange County | 52.4 (45.5, 59.3) |
| Orleans County | Suppressed |
| Oswego County | Suppressed |
| Otsego County | Suppressed |
| Putnam County | Suppressed |
| Queens County | 46.9 (44.0, 49.7) |
| Rensselaer County | 63.4 (55.6, 71.1) |
| Richmond County | 48.9 (43.3, 54.6) |
| Rockland County | 43.6 (35.6, 51.5) |
| Saratoga County | 55.1 (47.6, 62.6) |
| Schenectady County | 51.9 (43.2, 60.7) |
| Schoharie County | Suppressed |
| Schuyler County | Suppressed |
| Seneca County | Suppressed |
| St. Lawrence County | 64.0 (55.1, 72.8) |
| Steuben County | Suppressed |
| Suffolk County | 49.0 (45.5, 52.5) |
| Sullivan County | Suppressed |
| Tioga County | Suppressed |
| Tompkins County | Suppressed |
| Ulster County | 51.1 (43, 59.1) |
| Warren County | Suppressed |
| Washington County | Suppressed |
| Wayne County | Suppressed |
| Westchester County | 42.6 (38.2, 46.9) |
| Wyoming County | Suppressed |
| Yates County | Suppressed |

Abbreviation: CI, Confidence Interval

^a^ Arthritis, asthma, cancer, chronic obstructive pulmonary disease, depression, diabetes, heart disease, high blood pressure, high cholesterol, kidney disease, obesity, stroke
