# Supplementary material for: Prevalence of multiple chronic conditions in New York State, 2011–2016
Source: PLoS One. 2019 Feb 7;14(2):e0211965. doi: 10.1371/journal.pone.0211965 (PMC6366719; doi:10.1371/journal.pone.0211965)
Supplement: S2 Table — Behavioral Risk Factor Surveillance System, 2011–2016. (DOCX) [file pone.0211965.s002.docx]

# Supplementary Table 2. Prevalence of New York City Adults with Two or More Chronic Conditions^a^ by United Hospital Fund (UHF 42) Neighborhood. Behavioral Risk Factor Surveillance System, 2011–2016.

| **UHF Code** | **Neighborhood** | **Borough** | **% (95% CI)** |
| --- | --- | --- | --- |
| 101 | Kingsbridge - Riverdale | Bronx | 43.9 (34.0, 53.9) |
| 102 | Northeast Bronx | Bronx | 40.2 (31.0, 49.4) |
| 103 | Fordham - Bronx Park | Bronx | 53.8 (45.2, 62.3) |
| 104 | Pelham - Throgs Neck | Bronx | 49.5 (41.6, 57.3) |
| 105 | Crotona - Tremont | Bronx | Suppressed |
| 106 | High Bridge - Morrisania | Bronx | 60.6 (50.8, 70.4) |
| 107 | Hunts Point - Mott Haven | Bronx | Suppressed |
| 201 | Greenpoint | Brooklyn | Suppressed |
| 202 | Downtown - Heights - Park Slope | Brooklyn | 38.5 (32.2, 44.8) |
| 203 | Bedford Stuyvesant - Crown Heights | Brooklyn | 48.1 (41.2, 55.1) |
| 204 | East New York | Brooklyn | Suppressed |
| 205 | Sunset Park | Brooklyn | Suppressed |
| 206 | Borough Park | Brooklyn | 41.0 (32.7, 49.4) |
| 207 | East Flatbush - Flatbush | Brooklyn | 46.8 (39.5, 54.1) |
| 208 | Canarsie - Flatlands | Brooklyn | 48.2 (39.1, 57.4) |
| 209 | Bensonhurst - Bay Ridge | Brooklyn | Suppressed |
| 210 | Coney Island - Sheepshead Bay | Brooklyn | 46.2 (37.0, 55.5) |
| 211 | Williamsburg - Bushwick | Brooklyn | Suppressed |
| 301 | Washington Heights - Inwood | Manhattan | 48.9 (40.8, 57.0) |
| 302 | Central Harlem - Morningside Heights | Manhattan | 57.3 (48.5, 66.2) |
| 303 | East Harlem | Manhattan | Suppressed |
| 304 | Upper West Side | Manhattan | 39.1 (33.1, 45.1) |
| 305 | Upper East Side | Manhattan | 37.7 (31.6, 43.8) |
| 306 | Chelsea - Clinton | Manhattan | 44.2 (36.2, 52.2) |
| 307 | Gramercy Park - Murray Hill | Manhattan | 33.5 (26.0, 41.1) |
| 308 | Greenwich Village - SoHo | Manhattan | Suppressed |
| 309 | Union Square - Lower East Side | Manhattan | 43.1 (34.7, 51.5) |
| 310 | Lower Manhattan | Manhattan | Suppressed |
| 401 | Long Island City - Astoria | Queens | 41.3 (32.3, 50.4) |
| 402 | West Queens | Queens | 48.3 (40.9, 55.6) |
| 403 | Flushing - Clearview | Queens | 47.9 (39.3, 56.5) |
| 404 | Bayside - Little Neck | Queens | Suppressed |
| 405 | Ridgewood - Forest Hills | Queens | 44.7 (36.2, 53.2) |
| 406 | Fresh Meadows | Queens | Suppressed |
| 407 | Southwest Queens | Queens | 46.1 (37.8, 54.4) |
| 408 | Jamaica | Queens | 44.4 (36.5, 52.4) |
| 409 | Southeast Queens | Queens | 48.1 (39.1, 57.1) |
| 410 | Rockaway | Queens | Suppressed |
| 501 | Port Richmond | Staten Island | Suppressed |
| 502 | Stapleton - St. George | Staten Island | Suppressed |
| 503 | Willowbrook | Staten Island | Suppressed |
| 504 | South Beach - Tottenville | Staten Island | 50.5 (41.9, 59.1) |

Abbreviation: CI, Confidence Interval

^a^ Arthritis, asthma, cancer, chronic obstructive pulmonary disease, depression, diabetes, heart disease, high blood pressure, high cholesterol, kidney disease, obesity, stroke
